# Associations of social support and disease-specific quality of life with self-management among patients with chronic hepatitis B: a multicenter cross-sectional study

**DOI:** 10.3389/fpubh.2026.1962656

**Published:** 2026-10-07

**Authors:** Wen-Ting Yi, Ling-Ling Li, Mao-Ting Huang, Hui-Ling Yan, Jia-Jun Liu, Yi-Jing Chen, Xiao-Yan Huang, Jing-Zhi Yang, Ke-Hao Zhao, Ying Zeng

**Affiliations:** 1 Department of International and Humanistic Nursing, Hunan Science Popularization Education Base, School of Nursing, Hengyang Medical School, University of South China, Hengyang, China; 2 The First Affiliated Hospital, Department of Gastroenterology, Hengyang Medical School, University of South China, Hengyang, Hunan, China; 3 Hunan Engineering Research Center for Early Diagnosis and Treatment of Liver Cancer, Hunan Province Key Laboratory of Tumor Cellular & Molecular Pathology, Cancer Research Institute, Hengyang Medical School, University of South China, Hengyang, China

**Keywords:** chronic hepatitis B, quality of life, self-management, social stigma, social support

## Abstract

**Background:**

Self-management behaviors are central to long-term care for chronic hepatitis B (CHB), but the independent associations of social support and disease-specific quality-of-life domains with self-management remain unclear. This study assessed self-management and its associated factors among patients with CHB.

**Methods:**

This multicenter cross-sectional study included 583 patients with CHB recruited by convenience sampling at three tertiary hospitals in Hengyang, China, from March to October 2025. Participants completed the Chronic Hepatitis B Self-Management Scale (CHBSMS), Social Support Rating Scale (SSRS), and Chronic Hepatitis B Quality of Life Instrument (CHBQOL). Hierarchical multiple linear regression, HC3 robust standard errors, and sensitivity analyses were used.

**Results:**

The mean total CHBSMS score was 85.03 ± 19.38. The sociodemographic model yielded an *R*^2^ of 0.194. Adding clinical and healthcare utilization factors increased the explained variance (Δ*R*^2^ = 0.083), and adding social support and the four CHBQOL domain scores further increased the explained variance (Δ*R*^2^ = 0.102), yielding a final *R*^2^ of 0.379. In the fully adjusted model, social support (*B* = 0.577, 95% CI: 0.416–0.739, *p* < 0.001), the emotional symptoms domain (*B* = 0.170, 95% CI: 0.039–0.301, *p* = 0.011), and the social stigma domain (*B* = 0.235, 95% CI: 0.082–0.388, *p* = 0.003) were positively associated with self-management scores. Higher scores in the latter two domains indicated fewer emotional symptoms and less social stigma, respectively. For a 1-SD higher value of each predictor, the corresponding CHBSMS differences were 4.79, 1.90, and 2.15 points for social support, emotional symptoms, and social stigma, respectively, equivalent to 4.79, 1.90, and 2.15% of the score range. The somatic symptoms and belief domains were not significantly associated with self-management. Robustness and sensitivity analyses did not materially alter the main findings.

**Conclusion:**

Among patients with CHB recruited from three tertiary hospitals, social support, the emotional symptoms domain, and the social stigma domain were associated with self-management after adjustment for sociodemographic, clinical, and healthcare utilization factors. Further longitudinal research is needed to clarify the temporal direction and clinical relevance of these associations.

## 1 Introduction

Chronic hepatitis B (CHB) is a persistent inflammatory liver disorder arising from prolonged hepatitis B virus (HBV) infection. The disease often spans decades, imposing substantial physical, psychological, and economic burden on affected individuals (1). In 2022, an estimated 254 million individuals worldwide were living with chronic HBV infection, and hepatitis B-related deaths approached 1.1 million, largely attributable to cirrhosis and hepatocellular carcinoma (2, 3). In China, around 75 million people carry HBV, representing nearly 30% of the global hepatitis B burden (4). Although substantial progress has been made through hepatitis B vaccination, prevention of mother-to-child transmission, and antiviral treatment, major challenges remain in long-term health management (5, 6).

Self-management refers to an individual’s capacity to manage the symptoms, treatment, physical and psychosocial consequences, and lifestyle adjustments inherent in chronic illness (7), and has been widely applied across diverse chronic disease populations (8, 9). For patients with CHB, disease-specific management requires sustained treatment adherence, regular follow-up and monitoring, lifestyle modification, and effective coping with disease-related psychological distress (1, 10). However, many patients with CHB have suboptimal levels of self-management and treatment adherence (11, 12).

Evidence to date suggests that self-management behaviors among patients with CHB are associated with sociodemographic, clinical, psychosocial, and cognitive factors (10, 11, 13). Among these factors, social support may serve as an important external psychosocial resource for long-term disease management. Support from family members, peers, and healthcare professionals has been linked to greater motivation for disease management, better treatment adherence, and more positive expectations about health (14, 15). In addition to social support, patients’ disease-specific experiences of CHB may also be relevant to long-term disease management (16, 17). Disease-specific health-related quality of life in CHB reflects not only patients’ overall health status but also their somatic symptoms, emotional symptoms, belief, and social stigma (18). Adverse experiences across these domains may be associated with lower willingness and confidence to engage in long-term disease-management tasks and poorer self-management (19–21).

Although social support and health-related quality of life have each been examined in relation to self-management among patients with CHB, few studies have simultaneously evaluated social support and individual disease-specific quality-of-life domains within the same multivariable model after adjustment for sociodemographic, clinical, and healthcare utilization factors (15, 22, 23). In addition, health-related quality of life has often been treated as an outcome of self-management interventions in patients with CHB (23, 24) or analyzed using an overall or composite score (25–27), leaving limited evidence on whether the somatic symptoms, emotional symptoms, belief, and social stigma domains are differentially associated with self-management. Finally, multicenter studies among Chinese patients with CHB remain scarce.

In this context, we conducted a multicenter cross-sectional study among patients with CHB. Using a hierarchical modeling strategy, we first examined sociodemographic factors, then added clinical and healthcare utilization factors, and finally evaluated the associations of social support and the four CHBQOL domain scores with self-management after adjustment for the preceding factors. We also tracked changes in explained variance across the sequential models. This approach may provide further insight into self-management and its associated factors, with implications for future longitudinal and intervention studies on long-term self-management support for patients with CHB.

## 2 Methods

### 2.1 Study design, setting, and participants

This study was reported in accordance with the Strengthening the Reporting of Observational Studies in Epidemiology (STROBE) guidelines for cross-sectional studies (28). Participant recruitment was conducted from March to October 2025 in hepatology-related outpatient and inpatient departments at three tertiary hospitals in Hengyang, China, using convenience sampling.

Participants were eligible if they: (1) were aged 18 years or older; (2) had a diagnosis of CHB according to the Chinese Guidelines for the Prevention and Treatment of Chronic Hepatitis B (2022 edition) (1); (3) were able to understand the study information and complete the questionnaire; and (4) provided electronic informed consent.

Patients were excluded if they: (1) had a severe psychiatric disorder that precluded questionnaire completion; or (2) had decompensated cirrhosis, advanced hepatocellular carcinoma, or another severe comorbidity that precluded participation in the survey.

The initial recruitment target was estimated using a formula for estimating a population mean (29) to ensure adequate precision for describing CHBSMS scores. Using a standard deviation of 14.85 from a previous study (11), an allowable error of 1.4, and a two-sided 95% confidence level, the minimum sample size was estimated as 433. Allowing for a 20% rate of invalid or incomplete questionnaires, the target sample size was 542. Because the primary analysis was hierarchical multiple linear regression, the minimum incremental effect size detectable with the final sample was further evaluated using a sensitivity power analysis conducted with G*Power version 3.1.9.7 (30). Using an F test for linear multiple regression (fixed model, *R*^2^ increase), with an achieved sample size of 583, five predictors tested in the final block, 27 predictors in the full model, *α* = 0.05, and power (1 − *β*) = 0.80, the minimum detectable incremental effect size was Cohen’s *f*^2^ = 0.022.

### 2.2 Variable selection and hierarchical modeling strategy

The analytical framework distinguished self-management behaviors as the primary outcome and potential associated factors. The selection of study variables was informed by previous studies of self-management among patients with CHB (10, 11, 31) and the Individual and Family Self-Management Theory (IFSMT) (32). The IFSMT emphasizes the roles of contextual and process-related factors in self-management. Potential associated factors were organized into three analytical blocks according to their conceptual attributes. Sociodemographic factors were entered first as relatively stable background characteristics, followed by clinical and healthcare utilization factors reflecting participants’ health status and healthcare experiences. These two blocks were considered contextual factors within the IFSMT. Social support was conceptually aligned with social facilitation within the process dimension, whereas the four CHBQOL domains captured disease-specific somatic, emotional, belief-related, and social experiences. Accordingly, social support and the CHBQOL domains were entered together in the final block as the primary psychosocial and disease-specific factors of interest, allowing their incremental associations with self-management to be examined after adjustment for the preceding factors.

### 2.3 Measures

#### 2.3.1 General information questionnaire

A researcher-developed general information questionnaire was used to collect sociodemographic, clinical, and healthcare utilization data. The variables included sex, age, educational level, residence, marital status, annual household income, medical payment method, smoking status, alcohol consumption, disease duration, antiviral treatment status, the number of outpatient visits in the past year, history of hypertension and diabetes.

#### 2.3.2 Chronic Hepatitis B Self-Management Scale (CHBSMS)

Self-management was assessed using the Chronic Hepatitis B Self-Management Scale (CHBSMS) developed by Kong et al. (33). The scale comprises 25 items distributed across four dimensions: symptom management (Items 1–6), lifestyle management (Items 7–13), psychosocial coping (Items 14–19), and disease information management (Items 20–25). Each item is rated on a 5-point Likert scale, with total scores ranging from 25 to 125. Higher scores indicate greater self-management behaviors. In the present study, Cronbach’s α was 0.88.

#### 2.3.3 Social Support Rating Scale (SSRS)

Social support was assessed using the Social Support Rating Scale (SSRS) developed by Xiao (34). The scale comprises 10 items across three domains: objective support (Items 2, 6, and 7; score range, 1–22), subjective support (Items 1, 3–5; score range, 8–32), and support utilization (Items 8–10; score range, 3–12). Items are scored using item-specific response formats, and the item scores are summed to yield a total score ranging from 12 to 66. Higher scores indicate greater social support. In the present study, Cronbach’s *α* was 0.81.

#### 2.3.4 Chronic Hepatitis B Quality of Life Instrument (CHBQOL)

Disease-specific health-related quality of life was assessed using the Chronic Hepatitis B Quality of Life Instrument (CHBQOL) developed by Zhu et al. (18). The instrument comprises 23 items across four domains: somatic symptoms (Items 1–6), emotional symptoms (Items 7–12), belief (Items 13–14), and social stigma (Items 15–23). Each item is rated on a six-point Likert-type scale ranging from 0 (“not at all”) to 5 (“very much”). Items 1–12 and 15–23 were reverse-scored. Item scores were summed within each domain and linearly transformed to a 0–100 scale, with higher scores indicating better disease-specific health-related quality of life. The four transformed domain scores were used in subsequent analyses. In the present study, Cronbach’s *α* was 0.81 for somatic symptoms, 0.87 for emotional symptoms, 0.77 for belief, and 0.84 for social stigma.

### 2.4 Data collection and quality control

Trained researchers administered the survey using standardized procedures. Eligible patients were informed about the study purpose, procedures, confidentiality, and their right to withdraw before providing consent and completing the questionnaire electronically. Researchers could clarify questionnaire instructions but did not influence participants’ responses. Submissions were checked on site for missing data, duplication, and logical inconsistencies, and invalid or substantially incomplete questionnaires were excluded. As shown in Figure 1, 691 patients were screened, of whom 624 were eligible; 18 declined participation and 606 completed the questionnaire. After excluding 23 incomplete questionnaires, 583 participants were included in the final analysis. The participation rate was 97.1%, and the valid questionnaire rate was 96.2%.

**Figure 1:**
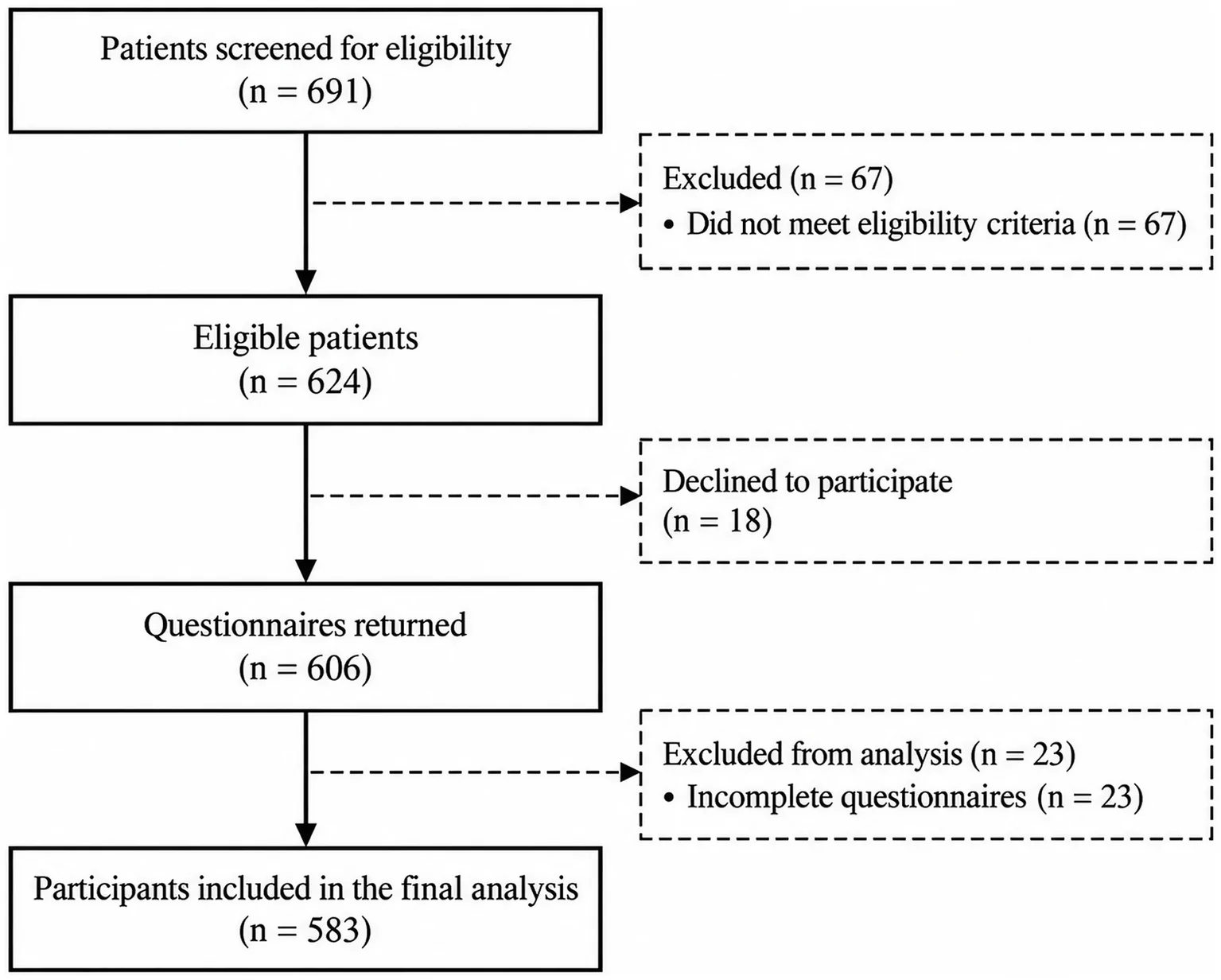
Flow diagram of participants through the study.

### 2.5 Statistical analysis

All statistical analyses were performed using R version 4.5.2. Categorical variables were presented as frequencies and percentages, and continuous variables as means and standard deviations. Differences in self-management scores across participant characteristics were examined using independent-samples *t* tests or one-way analysis of variance, as appropriate. Pearson’s correlations were calculated between self-management scores and social support and the four CHBQOL domains, as well as between the four CHBSMS dimensions and four CHBQOL domains to assess potential construct overlap. Potential common method variance was assessed using Harman’s single-factor test across all CHBSMS, SSRS, and CHBQOL items.

Hierarchical multiple linear regression analysis was performed with the CHBSMS total score as the dependent variable. Sociodemographic variables were entered in Model I; clinical and healthcare utilization variables were added in Model II; and social support, measured using the SSRS total score, and the four CHBQOL domain scores were added in Model III. Categorical predictors were dummy-coded, with the lowest or most clinically relevant category used as the reference group. Alcohol consumption was not included in the primary models because the CHBSMS contains an alcohol-related item, which could introduce content overlap between the covariate and the outcome.

Model assumptions and influential observations were assessed using residual diagnostics, the Shapiro–Wilk test, Breusch–Pagan test, variance inflation factors, externally studentized residuals, Cook’s distance, and leverage. Potentially influential observations were identified using an absolute externally studentized residual >3, Cook’s distance >4/*N*, or leverage >2*p/N*. Robustness and sensitivity analyses included HC3 robust standard errors; exclusion of potentially influential observations; additional adjustment for alcohol consumption; alternative CHBSMS and SSRS scores excluding the six psychosocial coping items and the support-utilization component, respectively, to assess potential content overlap; and reversal of the entry order of the clinical and healthcare utilization block and the social support/CHBQOL block, with sociodemographic factors retained in the first block. All statistical tests were two-sided, and *p* < 0.05 was considered statistically significant.

### 2.6 Ethical considerations

The study was approved by the Ethics Committee of the University of South China (approval no. 2024HLSC007). Electronic informed consent was obtained from all participants before questionnaire completion. Participation was voluntary, and responses were collected anonymously and kept confidential.

## 3 Results

### 3.1 Participant characteristics

Among 583 participants, 296 (50.8%) were male, and 287 (49.2%) were female. Overall, 53.9% of participants were aged 45–65 years, 81.5% were married, and 60.0% lived in rural areas. Self-management scores differed significantly by age, educational level, residence, annual household income, medical payment method, disease duration, and number of outpatient visits in the past year (all *p* < 0.05). Detailed characteristics and univariate analysis results are presented in Table 1.

**Table 1:** Participant characteristics and univariate analysis of self-management scores (*N* = 583).

Variables	Categories	*N* (%)	Score, mean ± SD	*t/F*	*p*
Sex	Male	296 (50.8)	85.38 ± 19.57	0.44^a^	0.661
	Female	287 (49.2)	84.67 ± 19.22		
Age (years)	<45	103 (17.7)	89.52 ± 18.48	4.14^b^	0.016
	45–65	314 (53.9)	84.85 ± 18.94		
	>65	166 (28.5)	82.59 ± 20.37		
Educational level	Primary school or below	138 (23.7)	76.93 ± 19.91	16.50^b^	<0.001
	Junior high school	224 (38.4)	84.97 ± 19.27		
	High school/technical secondary school	166 (28.5)	88.26 ± 17.49		
	College or above	55 (9.4)	95.86 ± 16.00		
Residence	Urban	233 (40.0)	88.23 ± 19.01	3.28^a^	0.001
	Rural	350 (60.0)	82.90 ± 19.37		
Marital status	Married	475 (81.5)	84.31 ± 19.37	−1.89^a^	0.059
	Unmarried/divorced/widowed	108 (18.5)	88.21 ± 19.23		
Annual household income (RMB)	<40,000	204 (35.0)	81.36 ± 18.62	10.99^b^	<0.001
	40,000–80,000	284 (48.7)	85.19 ± 19.40		
	>80,000	95 (16.3)	92.45 ± 18.97		
Medical payment method	Basic medical insurance for urban and rural residents	287 (49.2)	88.58 ± 17.54	10.02^b^	<0.001
	Urban employee basic medical insurance	129 (22.1)	84.57 ± 19.66		
	New Rural Cooperative Medical Scheme	108 (18.5)	81.61 ± 20.24		
	Self-pay	59 (10.1)	75.05 ± 21.44		
Smoking status	No	409 (70.2)	84.37 ± 20.11	−1.27^a^	0.204
	Yes	174 (29.8)	86.60 ± 17.52		
Alcohol consumption	No	402 (69.0)	84.72 ± 19.48	−0.59^a^	0.557
	Yes	181 (31.0)	85.74 ± 19.20		
Disease duration (years)	0–10	163 (28.0)	81.23 ± 18.65	5.97^b^	0.003
	11–20	243 (41.7)	85.10 ± 19.10		
	>20	177 (30.4)	88.44 ± 19.89		
Antiviral treatment	Yes	482 (82.7)	85.15 ± 19.63	0.31^a^	0.755
	No	101 (17.3)	84.49 ± 18.27		
Number of outpatient visits in the past year	0–4	293 (50.3)	81.59 ± 20.16	9.30^b^	<0.001
	5–8	176 (30.2)	85.89 ± 17.30		
	9–12	84 (14.4)	92.23 ± 19.76		
	>12	30 (5.1)	93.41 ± 13.36		
Hypertension	No	471 (80.8)	84.33 ± 19.42	−1.79^a^	0.073
	Yes	112 (19.2)	87.98 ± 19.02		
Diabetes	No	492 (84.4)	84.40 ± 19.40	−1.85^a^	0.065
	Yes	91 (15.6)	88.47 ± 19.06		

a Independent-samples *t* test.

b One-way ANOVA.

### 3.2 Self-management behaviors, social support, and quality of life scores

The mean total CHBSMS score was 85.03 ± 19.38, and the mean total SSRS score was 34.73 ± 8.30. The mean CHBQOL domain scores were 54.17 ± 11.47 for somatic symptoms, 54.20 ± 11.17 for emotional symptoms, 55.20 ± 20.79 for belief, and 55.98 ± 9.16 for social stigma. For the somatic symptoms and belief domains, the median (IQR) scores were 53.33 (46.67–63.33) and 60.00 (40.00–70.00), with observed ranges of 16.67–90.00 and 0.00–100.00, respectively. Higher CHBQOL scores indicated fewer symptoms or less impairment (Table 2).

**Table 2:** Scores of self-management behaviors, social support, and CHBQOL domains (*N* = 583).

Variables	Number of items	Possible range	Score, mean ± SD
CHBSMS total score	25	25–125	85.03 ± 19.38
Symptom management	6	6–30	20.67 ± 5.94
Lifestyle management	7	7–35	23.20 ± 5.15
Psychosocial coping	6	6–30	20.73 ± 6.02
Disease information management	6	6–30	20.43 ± 5.92
SSRS total score	10	12–66	34.73 ± 8.30
Objective support	3	1–22	10.00 ± 3.05
Subjective support	4	8–32	18.33 ± 4.36
Utilization of support	3	3–12	6.39 ± 2.04
CHBQOL domains			
Somatic symptoms	6	0–100	54.17 ± 11.47
Emotional symptoms	6	0–100	54.20 ± 11.17
Belief	2	0–100	55.20 ± 20.79
Social stigma	9	0–100	55.98 ± 9.16

CHBSMS, Chronic Hepatitis B Self-Management Scale; SSRS, Social Support Rating Scale; CHBQOL, Chronic Hepatitis B Quality of Life Instrument. The CHBQOL domain scores were linearly transformed to a 0–100 scale, with higher scores indicating better disease-specific health-related quality of life.

### 3.3 Correlations among self-management scores, social support, and quality of life

Pearson’s correlation analysis showed that self-management scores were positively correlated with social support (*r* = 0.335, *p* < 0.001), somatic symptoms domain (*r* = 0.160, *p* < 0.001), emotional symptoms domain (*r* = 0.233, *p* < 0.001), and social stigma domain (*r* = 0.228, *p* < 0.001), but not with the belief domain (*r* = 0.050, *p* > 0.05; Table 3). Dimension-level correlations between the four CHBSMS dimensions and the four CHBQOL domains were low (*r* = 0.032–0.236; Supplementary Table S1). Harman’s single-factor test identified 10 components with eigenvalues >1, with the first factor accounting for 24.69% of the total variance, below the commonly used 40% threshold. However, this finding does not rule out common method variance.

**Table 3:** Correlations among self-management behaviors, social support, and CHBQOL domains (*N* = 583).

Variables	1	2	3	4	5	6
1. Self-management behaviors	1					
2. Social support	0.335^***^	1				
3. Somatic symptoms	0.160^***^	0.086^*^	1			
4. Emotional symptoms	0.233^***^	0.170^***^	0.333^***^	1		
5. Belief	0.050	0.097^*^	0.101^*^	0.105^*^	1	
6. Social stigma	0.228^***^	0.108^**^	0.246^***^	0.361^***^	0.082^*^	1

Values are Pearson’s correlation coefficients. ^*^p < 0.05; ^**^p < 0.01; ^***^p < 0.001.

### 3.4 Hierarchical multiple linear regression analysis

Hierarchical multiple linear regression analysis was performed with the CHBSMS total score as the dependent variable (Table 4). Model I explained 19.4% of the variance in self-management scores (*R^2^* = 0.194, adjusted *R*^2^ = 0.176; *F* = 10.563, *p* < 0.001). Adding clinical and healthcare utilization variables in Model II increased the explained variance by 8.3 percentage points (Δ*R*^2^ = 0.083, Δ*F* = 7.112, *p* < 0.001), yielding an *R*^2^ of 0.277 and an adjusted *R*^2^ of 0.249. The subsequent addition of social support and the four CHBQOL domain scores in Model III increased the explained variance by a further 10.2 percentage points (Δ*R*^2^ = 0.102, Δ*F* = 18.205, *p* < 0.001). The final model accounted for 37.9% of the variance in self-management scores (adjusted *R*^2^ = 0.349). Changes in *R*^2^ across the three models are shown in Figure 2.

**Table 4:** Hierarchical multiple linear regression model of self-management scores (*N* = 583).

Variable	Model I			Model II			Model III		
	*B* (95% CI)	*β*	*p*	*B* (95% CI)	*β*	*p*	*B* (95% CI)	*β*	*p*
Constant	74.550 (67.806, 81.293)		<0.001	64.155 (56.365, 71.946)		<0.001	20.298 (7.637, 32.959)		0.002
Sociodemographic factors									
Age (reference = <45 years)									
45–65 years	−5.411 (−9.376, −1.445)	−0.139	0.008	−4.909 (−8.731, −1.087)	−0.126	0.012	−3.031 (−6.630, 0.567)	−0.078	0.099
>65 years	−7.924 (−12.300, −3.549)	−0.185	<0.001	−7.796 (−12.006, −3.586)	−0.182	<0.001	−6.247 (−10.185, −2.309)	−0.146	0.002
Sex (reference = male)									
Female	−0.346 (−3.228, 2.536)	−0.009	0.814	−0.323 (−3.097, 2.451)	−0.008	0.819	−0.110 (−2.725, 2.505)	−0.003	0.934
Educational level (reference = primary school or below)									
Junior high school	7.155 (3.374, 10.935)	0.180	<0.001	7.135 (3.504, 10.766)	0.179	<0.001	5.809 (2.413, 9.204)	0.146	<0.001
High school/technical secondary school	10.131 (6.113, 14.149)	0.236	<0.001	10.120 (6.260, 13.979)	0.236	<0.001	8.659 (5.052, 12.266)	0.202	<0.001
College or above	18.950 (13.399, 24.500)	0.286	<0.001	19.166 (13.817, 24.515)	0.289	<0.001	16.670 (11.654, 21.686)	0.252	<0.001
Residence (reference = urban)									
Rural	−5.987 (−8.943, −3.030)	−0.151	<0.001	−5.655(−8.493, −2.817)	−0.143	<0.001	−4.360 (−7.021, −1.698)	−0.110	0.001
Marital status (reference = married)									
Unmarried/divorced/widowed	3.242 (−0.486, 6.971)	0.065	0.088	2.962 (−0.618, 6.542)	0.059	0.206	1.530 (−1.816, 4.875)	0.031	0.370
Annual household income (RMB; reference = <40,000)									
40,000–80,000	2.297 (−0.913, 5.506)	0.059	0.160	1.985 (−1.094, 5.064)	0.051	0.206	1.987 (−0.890, 4.863)	0.051	0.175
>80,000	9.919 (5.597, 14.242)	0.189	<0.001	9.948 (5.782, 14.114)	0.190	<0.001	7.603 (3.684, 11.522)	0.145	<0.001
Medical payment method (reference = self-pay)									
Basic medical insurance for urban and rural residents	11.916 (6.928, 16.904)	0.308	<0.001	13.050 (8.260, 17.840)	0.337	<0.001	12.404 (7.906, 16.902)	0.320	<0.001
Urban employee basic medical insurance	8.162 (2.673, 13.650)	0.175	0.004	9.185 (3.915, 14.455)	0.197	<0.001	7.882 (2.958, 12.805)	0.169	0.002
New Rural Cooperative Medical Scheme	5.333 (−0.316, 10.982)	0.107	0.064	5.771 (0.330, 11.212)	0.116	0.038	4.329 (−0.752, 9.410)	0.087	0.095
Clinical and healthcare utilization factors									
Smoking status (reference = no)									
Yes				0.977 (−2.118, 4.071)	0.023	0.536	1.421 (−1.489, 4.332)	0.034	0.338
Disease duration (years; reference = 0–10)									
11–20				5.573 (2.192, 8.954)	0.142	0.001	5.686 (2.532, 8.840)	0.145	<0.001
>20				8.611 (4.982, 12.239)	0.204	<0.001	8.502 (5.122, 11.881)	0.202	<0.001
Antiviral treatment (reference = no)									
Yes				−0.521 (−4.171, 3.129)	−0.010	0.779	−1.157 (−4.584, 2.271)	−0.023	0.508
Hypertension (reference = no)									
Yes				3.905 (0.405, 7.405)	0.079	0.029	3.145 (−0.124, 6.413)	0.064	0.059
Diabetes (reference = no)									
Yes				2.908 (−0.927, 6.742)	0.054	0.137	2.634 (−0.940, 6.209)	0.049	0.148
Number of outpatient visits in the past year (reference = 0–4)									
5–8				3.651 (0.449, 6.853)	0.087	0.025	3.627 (0.637, 6.616)	0.086	0.018
9–12				10.894 (6.759, 15.028)	0.198	<0.001	9.682 (5.815, 13.549)	0.176	<0.001
>12				10.246 (3.803, 16.689)	0.117	0.002	10.067 (4.068, 16.067)	0.115	0.001
Social support and CHBQOL domain scores									
Social support							0.577 (0.416, 0.739)	0.247	<0.001
CHBQOL domain scores									
Somatic symptoms							0.041 (−0.082, 0.163)	0.024	0.516
Emotional symptoms							0.170 (0.039, 0.301)	0.098	0.011
Belief							0.000 (−0.063, 0.064)	0.000	0.989
Social stigma							0.235 (0.082, 0.388)	0.111	0.003
*R* ^2^	0.194			0.277			0.379		
Adjusted *R*^2^	0.176			0.249			0.349		
Δ*R*^2^	0.194			0.083			0.102		
*F* or Δ*F* (*p*)	10.563 (<0.001)			7.112 (<0.001)			18.205 (<0.001)		

*B*, unstandardized coefficient; *β*, standardized coefficient; *R*^2^, coefficient of determination; adjusted *R*^2^, adjusted coefficient of determination; Δ*R*^2^, change in *R*^2^ from the preceding model. Hierarchical entry: Model I included sociodemographic variables; Model II further added clinical and healthcare utilization variables; Model III additionally incorporated social support and the four CHBQOL domains. Alcohol consumption was excluded from the primary model because of direct content overlap with a CHBSMS alcohol-related item. *F* represents the overall model *F* statistic for Model I, whereas Δ*F* represents the *F*-change statistic for Models II and III.

**Figure 2:**
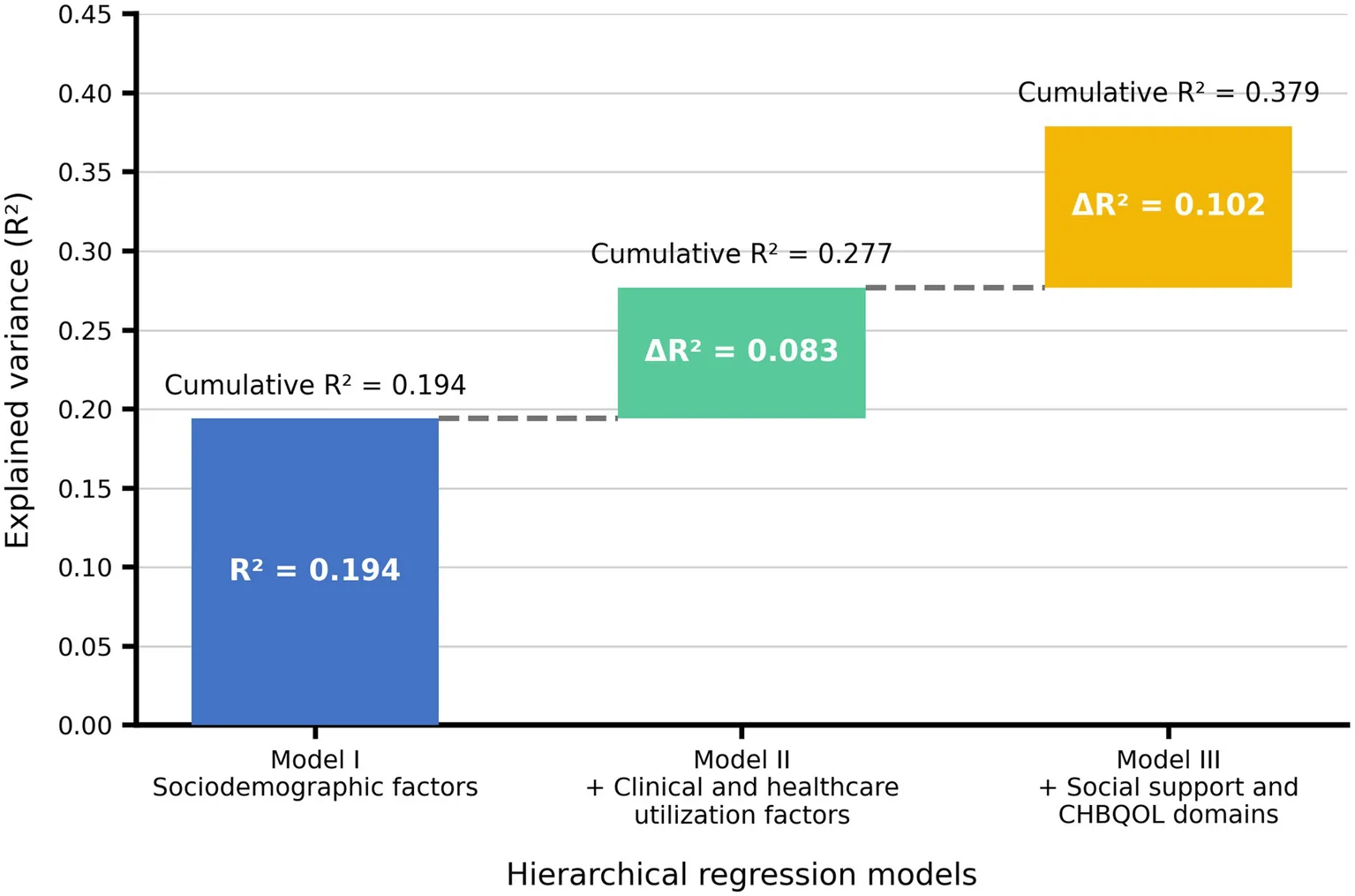
Incremental explained variance across the hierarchical regression models. Model I included sociodemographic factors. Model II additionally included clinical and healthcare utilization factors, and Model III further included social support and the four CHBQOL domain scores. Values within the bars indicate *R*^2^ for Model I and changes in *R*^2^ for Models II and III; values above the bars indicate cumulative *R*^2^. CHBQOL, Chronic Hepatitis B Quality of Life Instrument.

In the fully adjusted model, age >65 years and rural residence were associated with lower self-management scores. Education above primary school, an annual household income >80,000 RMB, coverage by basic medical insurance for urban and rural residents or urban employee basic medical insurance, a disease duration >10 years and ≥5 outpatient visits for hepatitis B in the past year were associated with higher self-management scores. After adjustment for these factors, social support (*B* = 0.577, 95% CI: 0.416–0.739, *β* = 0.247, *p* < 0.001), emotional symptoms (*B* = 0.170, 95% CI: 0.039–0.301, *β* = 0.098, *p* = 0.011), and social stigma (*B* = 0.235, 95% CI: 0.082–0.388, *β* = 0.111, *p* = 0.003) remained positively associated with self-management. A 1-SD higher social support score was associated with a 4.79-point higher CHBSMS score. The corresponding differences were 1.90 points for the emotional symptoms domain and 2.15 points for the social stigma domain. These differences represented 4.79, 1.90, and 2.15% of the full 100-point possible range of the CHBSMS. Somatic symptoms and belief were not significantly associated with self-management.

### 3.5 Regression diagnostics and sensitivity analyses

Residual plots showed no marked departure from linearity, and the residuals were approximately normally distributed (Shapiro–Wilk *W* = 0.995, *p* = 0.076). There was no evidence of heteroscedasticity (Breusch–Pagan LM = 31.210, *p* = 0.263), and the maximum variance inflation factor was 3.127. Two observations had absolute externally studentized residuals greater than 3, 21 exceeded the Cook’s distance criterion of 4/*N* (0.0069), and two exceeded the leverage criterion of 2*p/N* (0.0961), yielding 23 unique potentially influential observations. The maximum Cook’s distance was 0.023, and none exceeded 0.5. Excluding these observations did not materially change the core associations (Supplementary Figures S1–S5, Supplementary Table S2).

Sensitivity analyses yielded consistent results. The core associations remained materially unchanged with HC3 robust standard errors and after additional adjustment for alcohol consumption, which was not independently associated with self-management (*B* = 1.222, 95% CI: −1.588 to 4.031, *β* = 0.029, *p* = 0.393; Δ*R*^2^ = 0.001; Supplementary Table S2). They also remained consistent after excluding the six CHBSMS psychosocial coping items (social support: *β* = 0.243, *p* < 0.001; emotional symptoms: *β* = 0.087, *p* = 0.028; social stigma: *β* = 0.118, *p* = 0.002; Supplementary Table S3) and the SSRS support-utilization component (*β* = 0.245, *p* < 0.001; *R*^2^ = 0.378; Supplementary Table S4). Reversing the order of the latter two predictor blocks produced the same final model fit (*R*^2^ = 0.379, adjusted *R*^2^ = 0.349; Supplementary Table S5).

## 4 Discussion

The findings showed that self-management in CHB was associated with sociodemographic, clinical, healthcare utilization, psychosocial, and disease-specific quality-of-life factors. The hierarchical models further indicated that social support and CHBQOL domains explained additional variance beyond sociodemographic, clinical, and healthcare utilization factors. These results highlight the multifactorial nature of self-management in patients with CHB.

### 4.1 Level of self-management behaviors among patients with CHB

In our study, the mean total self-management score was 85.03 ± 19.38, which was approximately 68.0% of the total possible score. This was higher than the self-management score reported in a previous study of patients with CHB (74.64 ± 14.85; 59.7% of the total possible score) (11). It was also higher than the levels reported among rural Chinese patients with CHB, all of which were below 60% (31). The higher score observed in our study may reflect differences in sample composition, recruitment context, and healthcare settings across studies. However, the available data do not allow the reasons for this variation to be determined. Further studies using comparable populations and sampling procedures are needed to clarify these differences.

### 4.2 Sociodemographic and clinical correlates of self-management

In the fully adjusted model, participants aged >65 years and those living in rural areas had lower self-management scores, whereas higher educational attainment, higher annual household income, and medical insurance coverage were associated with higher scores. This pattern was broadly consistent with previous evidence (31, 35). Older adults and rural residents may encounter more difficulty understanding health information, accessing specialist care, and maintaining regular follow-up, whereas patients with higher educational attainment and greater financial resources may be better able to understand long-term treatment requirements and cover ongoing medication and monitoring costs (36–38). In addition, longer disease duration and more frequent outpatient visits were associated with higher self-management scores, in line with evidence from CHB and other chronic disease populations (35, 39–41). These associations may be related to greater familiarity with disease management over time and more opportunities to receive professional guidance through repeated outpatient contact. Because the study was cross-sectional, the direction of these associations cannot be determined; patients with better self-management may also be more likely to maintain regular follow-up.

### 4.3 Social support in relation to self-management behaviors

Social support showed a positive correlation with self-management and retained this association after adjustment for sociodemographic, clinical, healthcare utilization, and CHBQOL domain variables. It also had the largest standardized coefficient among the variables added in Model III (*β* = 0.247, *p* < 0.001). Our findings are consistent with previous studies showing that greater social support is associated with better self-management in chronic disease populations (42–44). Patients may draw on support from different sources in sustaining their disease-management activities. For example, family members can provide routine reminders and practical assistance (45); peer support may provide shared experiences and emotional understanding for patients experiencing anxiety, self-isolation, and loneliness (46); and community and primary healthcare professionals can provide professional guidance through regular follow-up, health education, and coordinated referrals (47). It should be noted that the presence of social relationships does not necessarily translate into effective support; its value may depend on whether it meets patients’ actual needs and can be effectively used. In addition, the SSRS support-utilization component and the CHBSMS disease information management dimension are conceptually adjacent because both may involve seeking and using external resources. However, support utilization concerns the mobilization of social resources, whereas disease information management reflects disease-specific self-management behavior. The association between social support and self-management remained essentially unchanged when the support-utilization component was excluded, indicating that the observed association was not dependent on this component.

### 4.4 Differential associations of CHBQOL domains with self-management

An important finding of our study was that the associations between the CHBQOL domains and self-management were not uniform. Correlation analysis showed positive associations for the somatic symptoms, emotional symptoms, and social stigma domains, but not for the belief domain. In the fully adjusted model, only the emotional symptoms and social stigma domains remained associated with self-management, supporting a domain-specific interpretation.

For the emotional symptoms domain, higher scores remained positively associated with self-management scores in the fully adjusted model, indicating that fewer emotional symptoms were associated with better self-management. Previous studies have linked greater psychological distress to poorer self-management (10) and more severe depressive symptoms to less favorable medication-adherence trajectories (13). One possible explanation is that persistent emotional distress may coexist with heightened perceptions of disease-related threat and uncertainty, together with lower confidence and motivation for long-term disease management. Conversely, better self-management may be accompanied by greater confidence in managing the disease and better emotional well-being (48, 49), suggesting a potentially bidirectional association between emotional status and self-management. Similarly, higher scores on the social stigma domain remained positively associated with self-management scores in the final model, indicating that less social stigma was associated with better self-management. This result aligns with previous findings (50). Stigma may be associated with self-management through both anticipated social judgment and internalized negative self-perceptions. In the context of public misconceptions about HBV transmission, patients with CHB may fear rejection in family, intimate, or workplace settings. These concerns have been linked to concealment of infection status, limited disease-related communication, lower healthcare seeking, and poorer treatment adherence (51). Internalized stigma may involve shame, self-devaluation, and social withdrawal, and may also be related to less willingness to attend follow-up visits, seek health advice, or engage in other disease-management activities (50, 52).

These domain-specific associations should be interpreted with attention to both effect magnitude and potential measurement overlap. The associations of emotional symptoms and social stigma with self-management were statistically significant but modest, corresponding to approximately 2% of the CHBSMS scale range per 1-SD difference. As no validated minimal clinically important difference has been established for the CHBSMS, their clinical significance remains uncertain. Potential content overlap between the CHBSMS and CHBQOL should also be considered. Although both instruments contain psychosocial content, they assess different aspects of the patient experience. Correlations between the CHBSMS dimensions and CHBQOL domains were low (*r* = 0.032–0.236), and both associations remained significant after removing the six psychosocial coping items from the CHBSMS. Direct content overlap is unlikely to fully explain the observed associations.

Interestingly, the somatic symptoms domain was not statistically associated with self-management in the fully adjusted model. This finding differs from evidence in some other chronic conditions, in which greater symptom burden has been associated with poorer self-management outcomes (53, 54). One possible explanation is that the physical burden of CHB is stage-dependent, with physical functioning relatively preserved in non-advanced disease and becoming more impaired as liver disease progresses (55). As patients with decompensated cirrhosis or advanced hepatocellular carcinoma were excluded, somatic symptoms may have played a less prominent role in self-management in this sample. The association observed in the correlation analysis was no longer significant after adjustment for other factors, suggesting that it may have reflected the combined influence of disease severity, emotional status, or other background factors. Finally, the belief domain was not statistically associated with self-management scores in the fully adjusted model. This null finding should be interpreted cautiously. This domain contains only two items and showed relatively lower test–retest reliability in the original validation study (18), although internal consistency was acceptable in the present study (Cronbach’s *α* = 0.77). These measurement limitations may restrict its ability to capture broader belief-related experiences, and the null finding is therefore inconclusive with respect to the role of beliefs in self-management. Future studies using more comprehensive measures of belief-related constructs are needed to clarify their relationship with self-management behaviors.

### 4.5 Reverse causality and alternative explanations

Given the cross-sectional design of this study, the temporal ordering of social support, disease-specific quality of life, and self-management cannot be established. The order of variables in the hierarchical regression reflected the prespecified analytical framework rather than an assumed temporal sequence, and reciprocal or alternative pathways remain possible. Greater social support may be associated with better self-management, while patients with better self-management may also be more likely to seek or use available support. A similar bidirectional relationship is plausible for emotional symptoms. For social stigma, the present data cannot determine whether lower perceived stigma precedes better self-management or whether the observed association reflects other underlying differences between patients. These findings should therefore be interpreted as adjusted cross-sectional associations rather than causal relationships.

### 4.6 Implications

These findings may inform considerations for the long-term management of patients with CHB. First, self-management support may need to account for differences in age, education, residence, and financial resources, with clear health education and appropriate links to specialist care. Because health literacy was not assessed, future educational strategies should consider patients’ ability to understand and use health information and be evaluated across different levels of health literacy. Second, self-management support could be incorporated into routine follow-up and broader CHB prevention and control activities, with ongoing rather than one-time guidance. Third, routine CHB care could include attention to the social support available to patients and how effectively it can be used. Finally, the associations with emotional symptoms and social stigma suggest that psychosocial concerns should not be overlooked in long-term CHB care. Non-stigmatizing communication, privacy protection, access to psychological support where appropriate, and community education addressing misconceptions about HBV transmission may be considered. Prospective studies are needed to determine whether these approaches improve self-management and related health outcomes.

### 4.7 Strengths and limitations

This multicenter cross-sectional study used hierarchical regression to examine the additional variance explained by social support and CHBQOL domains beyond sociodemographic, clinical, and healthcare utilization factors. Analyzing the four CHBQOL domains separately, rather than using an overall quality-of-life score, allowed domain-specific associations with self-management to be examined. Model diagnostics and multiple robustness and sensitivity analyses supported the stability of the findings.

Several limitations should be considered. First, the cross-sectional design precluded determination of temporal sequence or causal relationships. Second, participants were recruited by convenience sampling from three tertiary hospitals in one city, and 82.7% were receiving antiviral treatment, which may limit generalizability to untreated patients or those not engaged in specialist care. Third, the observed variability in CHBSMS scores was greater than assumed in the initial sample-size calculation, resulting in lower precision for the estimated mean CHBSMS score than originally planned. Fourth, the main variables were assessed using self-administered questionnaires at the same time point, which may have introduced self-report and common method bias. Fifth, some degree of conceptual overlap between the measures cannot be fully excluded. Potential overlap was most relevant to the CHBSMS psychosocial coping items and the CHBQOL emotional symptoms and social stigma domains, as well as the SSRS support-utilization component and the CHBSMS disease information management dimension. Sixth, the CHBQOL belief domain contains only two items and showed modest test–retest reliability in the original validation study, limiting interpretation of the null finding. Finally, health literacy was not assessed, limiting interpretation of the associations with educational attainment and residence. Other potentially relevant factors, including self-efficacy, validated measures of depression and anxiety, and HBV-specific virological and liver disease severity indicators, were also not assessed and may have contributed to residual confounding.

## 5 Conclusion

From a domain-specific perspective, this study showed that self-management among patients with CHB was associated with sociodemographic, clinical, healthcare utilization, and psychosocial factors. In the fully adjusted model, higher social support, fewer emotional symptoms, and less social stigma were associated with higher self-management scores, whereas the somatic symptoms and belief domains were not significantly associated. The null finding for the belief domain should be interpreted cautiously given its limited measurement properties. Given the cross-sectional design, future longitudinal and intervention research should clarify the temporal relationships and evaluate whether strategies addressing these factors can improve self-management and health outcomes.

## Data Availability

The raw data supporting the conclusions of this article will be made available by the authors, without undue reservation.
