# Supplementary material for: Associations of social support and disease-specific quality of life with self-management among patients with chronic hepatitis B: a multicenter cross-sectional study
Source: Front Public Health. 2026 Oct 7;14:1962656. doi: 10.3389/fpubh.2026.1962656 (PMC13683648; doi:10.3389/fpubh.2026.1962656)
Supplement: Supplementary file 1 [file Data_Sheet_1.docx]

**Supplementary Material**

**Figure S1.** Residuals versus fitted values.

**Figure S2.** Normal Q-Q plot.

**Figure S3.** Scale-location plot.

**Figure S4.** Residuals versus leverage.

**Figure S5.** Cook’s distance.

**Table S1.** Dimension-level correlations between the CHBSMS and CHBQOL domains.

**Table S2.** Robustness and sensitivity analyses of the associations of social support and CHBQOL domains with self-management scores.

**Table S3.** Sensitivity analysis excluding the six psychosocial coping items from the CHBSMS.

**Table S4.** Sensitivity analysis excluding the utilization of support component from the SSRS.

**Table S5.** Sensitivity analysis using an alternative entry order of predictor blocks.

**Table S6.** Sensitivity analyses for sparse categories and alternative categorizations of selected covariates .

**Figure S1.** Residuals versus fitted values.

The solid horizontal line represents a residual value of zero, and the curved line represents a locally weighted smoothing line.

**Figure S2.** Normal Q–Q plot.

The diagonal line represents the expected distribution of residuals under approximate normality.

**Figure S3.** Scale–location plot.

The square root of the absolute standardized residuals is plotted against the fitted values; the curved line represents a locally weighted smoothing line.

**Figure S4.** Residuals versus leverage.

The horizontal dashed lines indicate externally studentized residual thresholds of ±3, and the vertical dashed line indicates the leverage screening threshold of 2*p/N* (= 0.0961).

**Figure S5.** Cook’s distance.

The horizontal dashed line indicates the screening threshold of *4/N* (= 0.0069).

**Table S1. Dimension-level correlations between the CHBSMS and CHBQOL domains (N = 583)**

| **Variable** | **1** | **2** | **3** | **4** | **5** | **6** | **7** | **8** |
| --- | --- | --- | --- | --- | --- | --- | --- | --- |
| **1. Symptom management** | 1 |  |  |  |  |  |  |  |
| **2. Lifestyle management** | 0.663*** | 1 |  |  |  |  |  |  |
| **3. Psychosocial coping** | 0.666*** | 0.496*** | 1 |  |  |  |  |  |
| **4. Disease information management** | 0.708*** | 0.550*** | 0.567*** | 1 |  |  |  |  |
| **5. Somatic symptoms** | 0.142*** | 0.123** | 0.151*** | 0.122** | 1 |  |  |  |
| **6. Emotional symptoms** | **0.218***** | 0.169*** | **0.215***** | 0.177*** | 0.333*** | 1 |  |  |
| **7. Belief** | 0.051 | 0.032 | 0.039 | 0.045 | 0.101* | 0.105* | 1 |  |
| **8. Social stigma** | **0.236***** | 0.180*** | **0.184***** | 0.167*** | 0.246*** | 0.361*** | 0.082* | 1 |

**Note:** Values are Pearson’s correlation coefficients. Variables 1–4 are CHBSMS dimensions and variables 5–8 are CHBQOL domains. CHBSMS, Chronic Hepatitis B Self-Management Scale; CHBQOL, Chronic Hepatitis B Quality of Life Instrument. **P* < 0.05; ***P* < 0.01; ****P* < 0.001

**Table S2.** Robustness and sensitivity analyses of the associations of social support and CHBQOL domains with self-management scores

| **Predictor** | **Primary analysis OLS (N = 583)** | | | **HC3 robust inference (N = 583)** | | | **Excluding potentially influential observations (N = 560)** | | | **Additionally adjusting for alcohol consumption (N = 583)** | | |
| --- | --- | --- | --- | --- | --- | --- | --- | --- | --- | --- | --- | --- |
|  | **B (95% CI)** | **β** | ***P*** | **B (95% CI)** | **β** | ***P*** | **B (95% CI)** | **β** | ***P*** | **B (95% CI)** | **β** | ***P*** |
| **Social support** | 0.577 (0.416, 0.739) | 0.247 | <0.001 | 0.577 (0.414, 0.740) | 0.247 | <0.001 | 0.562 (0.410, 0.714) | 0.253 | <0.001 | 0.584 (0.422, 0.746) | 0.250 | <0.001 |
| **Somatic symptoms** | 0.041 (−0.082, 0.163) | 0.024 | 0.516 | 0.041 (−0.085, 0.166) | 0.024 | 0.525 | 0.001 (−0.112, 0.114) | 0.001 | 0.982 | 0.039 (−0.083, 0.162) | 0.023 | 0.528 |
| **Emotional symptoms** | 0.170 (0.039, 0.301) | 0.098 | 0.011 | 0.170 (0.039, 0.301) | 0.098 | 0.011 | 0.168 (0.047, 0.290) | 0.104 | 0.007 | 0.168 (0.036, 0.299) | 0.097 | 0.012 |
| **Belief** | 0.000 (−0.063, 0.064) | 0.000 | 0.989 | 0.000 (−0.063, 0.064) | 0.000 | 0.992 | 0.003 (−0.056, 0.062) | 0.004 | 0.917 | −0.001 (−0.064, 0.062) | −0.001 | 0.977 |
| **Social stigma** | 0.235 (0.082, 0.388) | 0.111 | 0.003 | 0.235 (0.075, 0.395) | 0.111 | 0.004 | 0.273 (0.131, 0.416) | 0.137 | <0.001 | 0.234 (0.081, 0.387) | 0.111 | 0.003 |
| **Alcohol consumption (Yes vs. No)** | — | | | | | | | | | 1.222 (−1.588, 4.031) | 0.029 | 0.393 |
| ***R*²** | 0.379 | | | 0.379 | | | 0.416 | | | 0.380 | | |
| **Adjusted *R*²** | 0.349 | | | 0.349 | | | 0.386 | | | 0.348 | | |
| **Maximum VIF** | 3.127 | | | 3.127 | | | 3.323 | | | 3.133 | | |

**Note:** B, unstandardized regression coefficient; β, standardized regression coefficient; CI, confidence interval; CHBQOL, Chronic Hepatitis B Quality of Life Instrument; HC3, heteroscedasticity-consistent covariance matrix estimator; OLS, ordinary least squares; VIF, variance inflation factor.

All models adjusted for the same sociodemographic, clinical, healthcare utilization, social support, and CHBQOL variables; the alcohol-adjusted model additionally included alcohol consumption (reference: no alcohol consumption). The influence analysis excluded 23 unique observations meeting at least one criterion: an absolute externally studentized residual >3, Cook’s distance >*4/N*, or leverage >2*p/N*. Two observations met the studentized-residual criterion, 21 met the Cook’s distance criterion, and two met the leverage criterion; these criteria were not mutually exclusive. The maximum Cook’s distance was 0.023, and no observation had a Cook’s distance >0.5.

**Table S3.** Sensitivity analysis excluding the six psychosocial coping items from the CHBSMS

| **Predictor** | **Primary analysis**  **(25-item CHBSMS total score)** | | | **Sensitivity analysis**  **(19-item CHBSMS score)** | | |
| --- | --- | --- | --- | --- | --- | --- |
|  | **B (95% CI)** | **β** | ***P*** | **B (95% CI)** | **β** | ***P*** |
| **Social support** | 0.577 (0.416, 0.739) | 0.247 | <0.001 | 0.434 (0.308, 0.561) | 0.243 | <0.001 |
| **Somatic symptoms** | 0.041 (−0.082, 0.163) | 0.024 | 0.516 | 0.025 (−0.071, 0.122) | 0.020 | 0.604 |
| **Emotional symptoms** | 0.170 (0.039, 0.301) | 0.098 | 0.011 | 0.115 (0.012, 0.219) | 0.087 | 0.028 |
| **Belief** | 0.000 (−0.063, 0.064) | 0.000 | 0.989 | 0.003 (−0.047, 0.052) | 0.004 | 0.909 |
| **Social stigma** | 0.235 (0.082, 0.388) | 0.111 | 0.003 | 0.191 (0.071, 0.311) | 0.118 | 0.002 |
| **Model fit** | | | | | | |
| ***R*²** | **0.379** | | | **0.347** | | |
| **Adjusted *R*²** | **0.349** | | | **0.315** | | |

**Note:** B, unstandardized regression coefficient; CI, confidence interval; β, standardized regression coefficient; CHBSMS, Chronic Hepatitis B Self-Management Scale; CHBQOL, Chronic Hepatitis B Quality of Life Instrument. The primary outcome was the 25-item CHBSMS total score. The 19-item CHBSMS score excluded the six psychosocial coping items and included the symptom management, lifestyle management, and disease information management domains (range, 19–95; mean ± SD, 64.30 ± 14.86). Both models adjusted for the same covariates; N = 583.

**Table S4.** Sensitivity analysis excluding the utilization of support component from the SSRS

| **Predictor** | **Primary analysis (Full SSRS total score)** | | | **Sensitivity analysis (Objective + subjective support score)** | | |
| --- | --- | --- | --- | --- | --- | --- |
|  | **B (95% CI)** | **β** | ***P*** | **B (95% CI)** | **β** | ***P*** |
| **Social support** | 0.577 (0.416, 0.739) | 0.247 | <0.001 | 0.702 (0.504, 0.900) | 0.245 | <0.001 |
| **Somatic symptoms** | 0.041 (−0.082, 0.163) | 0.024 | 0.516 | 0.045 (−0.078, 0.167) | 0.027 | 0.471 |
| **Emotional symptoms** | 0.170 (0.039, 0.301) | 0.098 | 0.011 | 0.173 (0.042, 0.304) | 0.100 | 0.010 |
| **Belief** | 0.000 (−0.063, 0.064) | 0.000 | 0.989 | 0.001 (−0.062, 0.064) | 0.001 | 0.977 |
| **Social stigma** | 0.235 (0.082, 0.388) | 0.111 | 0.003 | 0.234 (0.080, 0.387) | 0.110 | 0.003 |
| **Model fit** | | | | | | |
| ***R*²** | **0.379** | | | **0.378** | | |
| **Adjusted *R*²** | **0.349** | | | **0.348** | | |

**Note:** In the primary analysis, social support was represented by the full SSRS total score (mean ± SD, 34.73 ± 8.30). In the sensitivity analysis, the full SSRS score was replaced by a reduced score comprising the objective and subjective support components only (mean ± SD, 28.33 ± 6.76), thereby excluding the utilization of support component. The full and reduced SSRS scores were highly correlated (r = 0.984). Both models were adjusted for the same covariates. N = 583.

**Table S5.** Sensitivity analysis using an alternative entry order of predictor blocks

| **Ordering** | **Model / variables entered** | ***R*²** | **Adjusted *R*²** | **Δ*R*²** | **ΔF** | ***P*** |
| --- | --- | --- | --- | --- | --- | --- |
| **Primary** | Model I: Sociodemographic factors | 0.194 | 0.176 | — | — | — |
|  | Model II: + Clinical and healthcare utilization factors | 0.277 | 0.249 | 0.083 | 7.112 | <0.001 |
|  | Model III: + Social support and CHBQOL domains | 0.379 | 0.349 | 0.102 | 18.205 | <0.001 |
| **Alternative** | Model I: Sociodemographic factors | 0.194 | 0.176 | — | — | — |
|  | Model II: + Social support and CHBQOL domains | 0.306 | 0.284 | 0.112 | 18.208 | <0.001 |
|  | Model III: + Clinical and healthcare utilization factors | 0.379 | 0.349 | 0.073 | 7.202 | <0.001 |

**Note:** CHBQOL, Chronic Hepatitis B Quality of Life Instrument; Δ*R*², change in explained variance; Δ*F*, F test for the change in *R*². The alternative model reversed the order of the latter two blocks while retaining sociodemographic factors in the first block. N = 583.

**Table S6.** Sensitivity analyses for sparse categories and alternative categorizations of selected covariates. (N = 583)

| **Predictor** | **Primary model β (*P*)** | **Sparse-category sensitivity β (*P*)** | **Broader-categorization sensitivity β (*P*)** |
| --- | --- | --- | --- |
| **Social support** | 0.247 (<0.001) | 0.256 (<0.001) | 0.267 (<0.001) |
| **Somatic symptoms** | 0.024 (0.516) | 0.024 (0.518) | 0.018 (0.628) |
| **Emotional symptoms** | 0.098 (0.011) | 0.094 (0.015) | 0.105 (0.008) |
| **Belief** | 0.000 (0.989) | 0.003 (0.928) | 0.000 (0.994) |
| **Social stigma** | 0.111 (0.003) | 0.113 (0.002) | 0.112 (0.003) |
| ***R*²** | **0.379** | **0.368** | **0.344** |
| **Adjusted *R*²** | **0.349** | **0.340** | **0.320** |

**Note: In the sparse-category sensitivity analysis,** ‘high school/technical secondary school’ and ‘college or above’ were combined as ‘high school or above,’ and ‘9–12 outpatient visits’ and ‘>12 visits’ were combined as ‘≥9 visits.’

**In the broader-categorization sensitivity analysis**, adjacent categories were further combined for age (<45 vs. ≥45 years), annual household income (<40,000 vs. ≥40,000 RMB), disease duration (0–10 vs. >10 years), and outpatient visit frequency (0–8 vs. ≥9 visits/year); educational level remained categorized as primary school or below, junior high school, and high school or above. No new cut-points were introduced. All other covariates and model specifications were unchanged from the primary fully adjusted model. β, standardized regression coefficient.
