# Supplementary material for: Associations of social support and disease-specific quality of life with self-management among patients with chronic hepatitis B: a multicenter cross-sectional study
Source: Front Public Health. 2026 Oct 7;14:1962656. doi: 10.3389/fpubh.2026.1962656 (PMC13683648; doi:10.3389/fpubh.2026.1962656)
Supplement: Supplementary file 2 [file Supplementary_file_1.docx]

# STROBE Statement—Checklist of items that should be included in reports of cross-sectional studies

| **Section and Item** | **Item No.** | **Recommendation** | Reported in |
| --- | --- | --- | --- |
| Title and abstract | 1(a) | Indicate the study’s design with a commonly used term in the title or the abstract | Title and Abstract |
| Title and abstract | 1(b) | Provide in the abstract an informative and balanced summary of what was done and what was found | Abstract |
| Introduction — Background/rationale | 2 | Explain the scientific background and rationale for the investigation being reported | Introduction |
| Introduction — Objectives | 3 | State specific objectives, including any prespecified hypotheses | Introduction |
| Methods — Study design | 4 | Present key elements of study design early in the paper | Methods — Section 2.1 |
| Methods — Setting | 5 | Describe the setting, locations, and relevant dates, including periods of recruitment, exposure, follow-up, and data collection | Methods — Section 2.1 |
| Methods — Participants | 6 | Give eligibility criteria, and the sources and methods of selection of participants | Methods — Sections 2.1 and 2.4; Figure 1 |
| Methods — Variables | 7 | Clearly define all outcomes, exposures, predictors, potential confounders, and effect modifiers | Methods — Sections 2.2–2.3 |
| Methods — Data sources/measurement | 8 | For each variable of interest, give sources of data and details of methods of assessment | Methods — Section 2.3 (Measures) |
| Methods — Bias | 9 | Describe any efforts to address potential sources of bias | Methods — Sections 2.4–2.5 |
| Methods — Study size | 10 | Explain how the study size was arrived at | Methods — Section 2.1 |
| Methods — Quantitative variables | 11 | Explain how quantitative variables were handled in the analyses. If applicable, describe which groupings were chosen and why | Methods — Sections 2.3.1 and 2.5; Tables 1 and 4; Supplementary Table S6 |
| Methods — Statistical methods | 12(a) | Describe all statistical methods, including those used to control for confounding | Methods — Section 2.5 (Statistical analysis) |
| Methods — Statistical methods | 12(b) | Describe any methods used to examine subgroups and interactions | N/A |
| Methods — Statistical methods | 12(c) | Explain how missing data were addressed | Methods — Section 2.4 (Data collection and quality control) |
| Methods — Statistical methods | 12(d) | If applicable, describe analytical methods taking account of sampling strategy | N/A |
| Methods — Statistical methods | 12(e) | Describe any sensitivity analyses | Methods — Section 2.5; Results — Section 3.5; Supplementary Material |
| Results — Participants | 13(a) | Report numbers of individuals at each stage of study | Methods — Section 2.4; Figure 1 |
| Results — Participants | 13(b) | Give reasons for non-participation at each stage | Methods — Section 2.4; Figure 1 |
| Results — Participants | 13(c) | Consider use of a flow diagram | Figure 1 |
| Results — Descriptive data | 14(a) | Give characteristics of study participants and information on exposures and potential confounders | Results — Section 3.1; Table 1 |
| Results — Descriptive data | 14(b) | Indicate number of participants with missing data for each variable of interest | Methods — Section 2.4 (incomplete questionnaires excluded) |
| Results — Outcome data | 15 | Report numbers of outcome events or summary measures | Results — Section 3.2; Table 2 |
| Results — Main results | 16(a) | Give unadjusted and adjusted estimates and their precision | Results — Sections 3.1, 3.3, and 3.4; Tables 1, 3, and 4 |
| Results — Main results | 16(b) | Report category boundaries when continuous variables were categorized | Methods — Section 2.3.1; Tables 1 and 4 |
| Results — Main results | 16(c) | If relevant, consider translating estimates of relative risk into absolute risk | N/A |
| Results — Other analyses | 17 | Report other analyses done, including sensitivity analyses | Results — Section 3.5; Supplementary Material |
| Discussion — Key results | 18 | Summarise key results with reference to study objectives | Discussion |
| Discussion — Limitations | 19 | Discuss limitations of the study, taking into account sources of potential bias or imprecision | Discussion — Section 4.7 (Strengths and limitations) |
| Discussion — Interpretation | 20 | Give a cautious overall interpretation of results considering objectives, limitations, multiplicity of analyses, results from similar studies, and other relevant evidence | Discussion — Sections 4.1–4.7 |
| Discussion — Generalisability | 21 | Discuss the generalisability (external validity) of the study results | Discussion — Section 4.7 (Strengths and limitations) |
| Other information — Funding | 22 | Give the source of funding and the role of funders for the present study | Funding |
